# Risk for multidrug-resistant tuberculosis in patients treated with anti-tumor necrosis factor agents

**DOI:** 10.3389/fmed.2023.1108119

**Published:** 2023-03-22

**Authors:** Jinkyeong Park, Yoonki Hong, Ji Young Hong

**Affiliations:** ^1^Department of Pulmonary, Allergy and Critical Care Medicine, Kyung Hee University Hospital at Gangdong, School of Medicine, Kyung Hee University, Seoul, Republic of Korea; ^2^Department of Internal Medicine, School of Medicine, Kangwon National University, Kangwon National University Hospital, Chuncheon, Republic of Korea; ^3^Division of Pulmonary, Allergy and Critical Care Medicine, Department of Internal Medicine, Chuncheon Sacred Heart Hospital, Hallym University Medical Center, Chuncheon, Gangwon-do, Republic of Korea

**Keywords:** multidrug-resistant tuberculosis, anti-TNF agents, infections, risk factor, pulmonary TB

## Abstract

**Background:**

There are few studies on medical conditions associated with the development of drug-resistant TB.

**Objective:**

We investigated the risk factors for the occurrence of multidrug-resistant (MDR) tuberculosis (TB) in patients with pulmonary TB.

**Materials and methods:**

Based on claims data from the Health Insurance Review and Assessment service in South Korea, we retrospectively investigated patients aged 18 years or older with active pulmonary TB who were treated with anti-TB therapy between January 1, 2008, and February 28, 2021.

**Results:**

Among 248,176 patients with pulmonary TB who underwent anti-TB therapy, 2.0% were identified as having MDR-TB. MDR-TB showed male predominance compared to patients without MDR-TB, and patients with MDR-TB were younger. The risk for MDR-TB in patients treated with anti-TB therapy was 3.26 times higher in patients who received anti-tumor necrosis factor (TNF) agents before prescription of anti-TB medications than in those who had never been exposed to anti-TNF agents after adjusting for other TB risk factors (age, sex, inhaled corticosteroid, diabetes mellitus, liver disease, pneumoconiosis, and organ or blood recipients). The risk for MDR-TB was also increased in males and younger patients.

**Conclusion:**

Treatment with an anti-TNF agent could be a driver of MDR-TB in patients with pulmonary TB.

## Background

The incidence rate of tuberculosis (TB) rose by 3.6% between 2020 and 2021, reversing years of decline for the previous 2 decades according to the 2022 WHO World Tuberculosis Report (1). Unfortunately, the burden of multidrug-resistant TB (MDR-TB) is also estimated to have increased between 2020 and 2021. Globally, the rate of MDR-TB among patients with new TB was about 3.6% and the best estimate for people who have previously been treated for TB is about 18% in 2021. Drug-susceptible TB and drug-resistant TB are spread in the same way (2). However, treatment of MDR-TB infections is much more complex, less effective, more toxic, and more expensive than treating patients infected with susceptible TB strains (3). Reducing the incidence of MDR-TB infection in the community may be difficult due to a lack of knowledge about the risk factors associated with developing MDR-TB.

The mechanisms that lead to the development of drug resistance are complex, and resistance may have multiple causes even within the same individual patient. Previous studies (4–7) have identified risk factors for developing anti-TB drug resistance. Some studies have suggested that drug resistance may be associated with prior exposure to anti-TB drugs in insufficient amounts to achieve complete cure (8, 9). Other studies have suggested that race, malnutrition, and pulmonary TB may be risk factors for developing drug-resistant TB (10). The onset of MDR-TB can be related to various factors, including race, socioeconomic status, culture, lifestyle, epidemiology, medical condition, and detection technology (10). Some comorbidity studies have shown that people with human immunodeficiency virus (HIV) infection or diabetes are more likely to develop MDR-TB (11–13).

However, few studies have identified the comorbidities or medical conditions associated with the development of drug-resistant TB (12, 13). Moreover, with the exception of HIV and diabetes, it is unclear whether the risk factors associated with TB already act as risk factors for developing drug-resistant TB (14, 15). We investigated the risk factors for the occurrence of MDR-TB in patients with pulmonary TB using a national population-based database.

## Materials and methods

### Study area and setting

This retrospective observational cohort study analyzed claims data from the Health Insurance Review and Assessment (HIRA) service in Korea between January 1, 2007, and February 28, 2021.

### Study design and study subjects

The study population consisted of patients aged 18 years or older diagnosed with TB (code 15–19, A30 of the International Classification of Diseases 10th revision: ICD-10) between July 1, 2007, and February 28, 2021, registered as officially designated infectious disease patients in Korea, and were continuously prescribed TB medications, such as isoniazid, rifampin, or ethambutol, for more than 2 months. Each patient was excluded if there was evidence of tuberculosis (disease code and prescription of tuberculosis-related medications) during the first-year baseline period to exclude retreatment and recurrence. Patients enrolled in this study were followed up for a median of 3,604 days [interquartile range (IQR) 2,950–4,229]. The exclusion criteria were as follows: age > 100 years; latent TB (code R76 of ICD-10); TB sequelae (B90 of ICD-10) or non-mycobacterium infection (code A31 of ICD-10); and infected with HIV (code B20–22, B24 of ICD-10).

In 2007, the Korean government extended health insurance coverage to patients with MDR-TB identified with code U88 or V206 in the NHI system. Patients were divided into two groups according to the time of onset: naïve MDR-TB defined as cases with an MDR code within 6 months of the start of TB treatment, and non-naïve MDR-TB in which it was assumed that MDR developed due to reinfection or *de novo* emergence. The anti-tumor necrosis factor (TNF) agents administered in the study population were infliximab, etanercept, and adalimumab.

### Data source

South Korea’s National Health Insurance (NHI) system is a public, single-payer system, which has covered the South Korean population since 1989. NHI covers 97% of the people, with the Medical Aid program covering the remaining 3%. The HIRA reviewed all claims data submitted by the NHI and the Medical Aid program.

### Ethics statement

The HIRA approved the research protocol (M20211011551). The requirement for ethics approval was waived by the Kangwon National University Hospital Institutional Review Board (KNUH-2022–02–011) because this study used only previously collected data. The study was performed in accordance with the Declaration of Helsinki.

### Statistical analysis

The primary endpoint was the incidence of MDR-TB with anti-TNF treatment. The secondary endpoints were the hazard ratios of anti-TNF agents for MDR-TB. The baseline variables and patient characteristics in each group are presented as percentages or the median [IQR]. Between-group comparisons were performed using the Mann–Whitney U test or chi-square test as appropriate. Kaplan–Meier curves allowed the comparison of MDR-TB incidence between groups using the log-rank test. Multivariate Cox regression analysis was performed to determine the hazard ratios for sex, age, and Charlson comorbidity index (CCI). All analyzes were carried out using R v.3.4.4, and *p* < 0.05 was taken to indicate statistical significance.

## Results

### Characteristics of study participants

During the study period, a total of 248,176 patients were diagnosed with TB and received anti-TB treatment. MDR-TB had a prevalence rate of 2.02% (*n* = 5,008) among patients with active TB (Table 1). The median time interval between onset of active TB and MDR-TB diagnosis was 161 [IQR 81–511] days and 52.8% (*n* = 2,642) of MDR-TB cases were naïve MDR-TB identified within 6 months of initial treatment with anti-TB drugs. Among the total population with TB, 23.54% had diabetes, and 4.74% (*n* = 11,769) had used an inhaled corticosteroid (ICS) before TB diagnosis; 0.70% had received an inhaled corticosteroid treatment within 1 year before diagnosis with TB. TB occurred at a median of 131 days [IQR 29–566.5] after the first use of an inhaled corticosteroid. Anti-TNF agents had been administered before the diagnosis of TB in 0.13% of patients with TB; the anti-TNF agents used were adalimumab (*n* = 147, 44.4%), infliximab (*n* = 123, 55.4%), and etanercept (*n* = 85, 34.0%). TB developed a median of 422 days [IQR 174–816] after first administration of anti-TNF agents (307 days [IQR 96–576] for infliximab, 312 days [IQR 167–572] for adalimumab, and 812 days [IQR 437–1,247] for etanercept).

**Table 1:** Baseline characteristics of patients with tuberculosis.

	All pulmonary TB	no MDR-TB	MDR-TB	value of *p*
No. of patients	248,176	243,168	5,008	
Female	100,215 (40.38)	98,566 (40.53)	1,649 (32.9)	<0.001
Age (years)^a^	53.0 [37.0, 71.0]	54.0 [37.0, 71.0]	46.0 [33. 0, 59. 0]	<0.001
CCI^a^	2.0 [1. 0, 5.0]	2.0 [1.0, 5.0]	2.0 [1.0, 4.0]	<0.001
No. of TB drugs used^a^	4.0 [4. 0, 4.0]	4.0 [4.0, 4.0]	7.0 [5.0, 8.0]	<0.001
DM	58,428 (23.54)	56,945 (23.42)	1,483 (29.6)	<0.001
Liver disease	92,779 (37.38)	91,100 (37.46)	1,679 (33.53)	<0.001
Pneumoconiosis	1,260 (0.51)	1,235 (0.51)	25 (0.51)	1
Solid organ transplant	486 (0.20)	479 (0.20)	7 (0.14)	0.456
BMT	64 (0.03)	63 (0.03)	1 (0.02)	1
Rheumatoid Arthritis	19,215 (7.74)	18,961 (7.80)	254 (5.07)	<0.001
Psoriasis	5,914 (2.38)	5,839 (2.40)	75 (0.16)	<0.001
Ankylosing Spondylitis	1996 (0.80)	1970 (0.81)	26 (0.52)	0.028
Crohn Disease	568 (0.23)	551 (0.23)	17 (0.34)	0.132
Ulcerative Colitis	1,173 (0.47)	1,156 (0.48)	17 (0.34)	0.199
ICS user	11,769 (4.74)	11,441 (4.70)	328 (6.55)	<0.001
ICS user within 1 year before TB diagnosis	1734 (0.70)	1706 (0.70)	28 (0.56)	0.266
Interval between ICS and TB (days)^a^	131. 0 [29.0, 566.5]	133.5 [30.0, 569.0]	36.5 [13.5, 189.8]	0.014
Tx with anti-TNF agent	312 (0.13)	298 (0.12)	14 (0.28)	0.004
Interval between anti-TNF and TB (days)^a^	421.5 [174.3,816.3]	420.5 [175.0, 808.0]	591.0 [168.8,1194.0]	0.398

MDR-TB, multidrug resistant tuberculosis; anti-TNF, anti-tumor necrosis factor; BMT, bone marrow transplantation; CCI, Charlson Comorbidities index; DM, diabetes mellitus; ICS, inhaled corticosteroid; No, number; Tx, treatment; TB, tuberculosis.

a Data are expressed in median [interquartile range].

MDR-TB showed male predominance compared to patients without MDR-TB (67.1% vs. 59.5%, respectively, *p* < 0.01), and patients with MDR-TB were younger (median age: 46 years [IQR 33–60] vs. 54 years [IQR 37–71], respectively, *p* < 0.01). Patients with MDR-TB had a higher incidence rate of diabetes mellitus and lower rates of liver disease, rheumatoid arthritis, ankylosing spondylosis and psoriasis than those without MDR-TB. In addition, MDR-TB patients had higher treatment rates with ICS (6.5% vs. 4.7%, *p* < 0.01, respectively) and anti-TNF agents (0.3% vs. 0.1%, *p* < 0.01, respectively) before diagnosis. MDR-TB developed after 559 days [IQR 303–1,146] in 2366 patients with non- naïve MDR-TB. There was no difference between naïve MDR-TB patients and non- naïve MDR-TB patients except that there were significantly more males and ICS users in non-naïve MDR-TB patients.

### Risk for MDR in patients receiving anti-TNF agents

**Figure 1:**
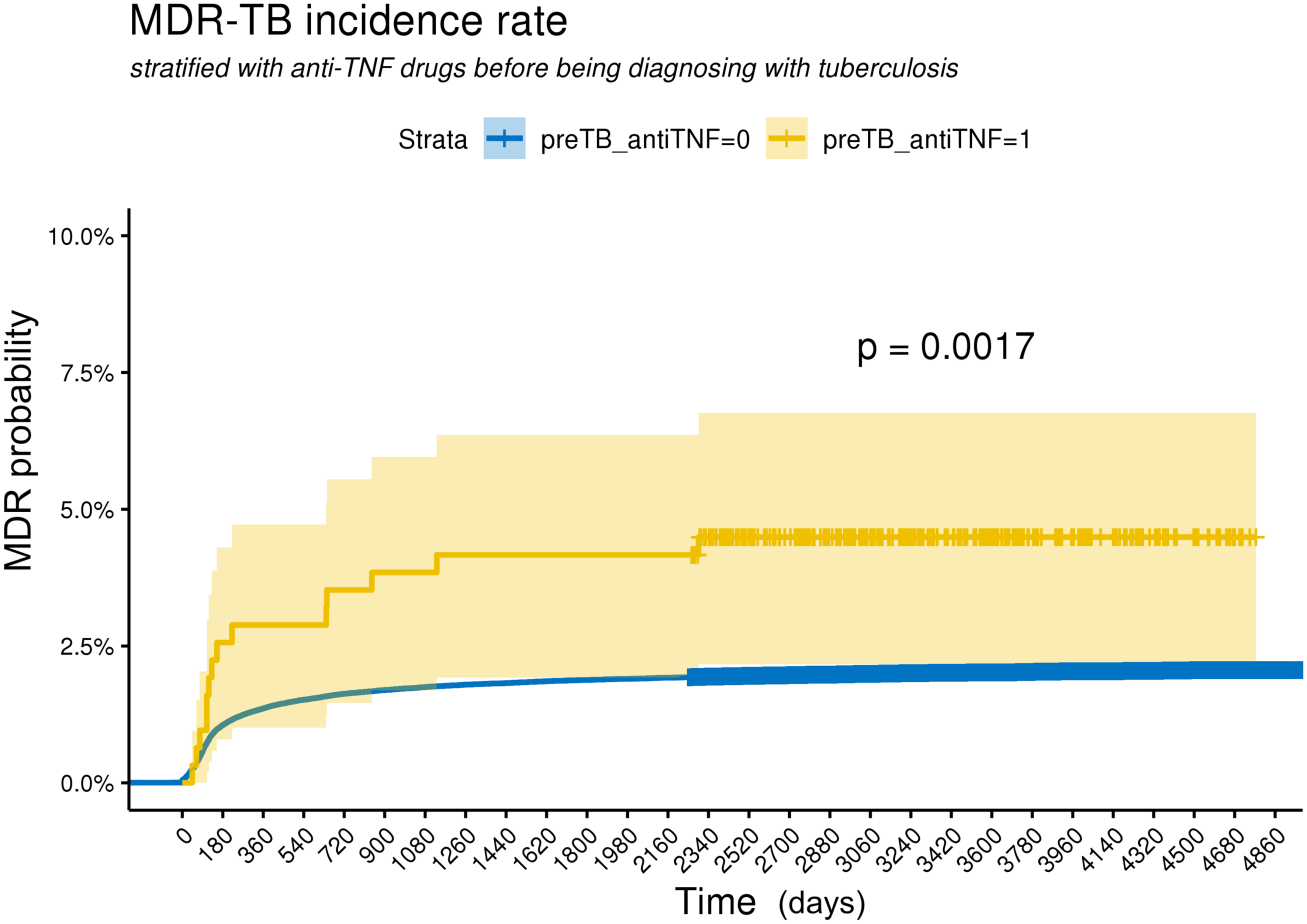
Kaplan Meier curves for cumulative probability of MDR-TB according to the use with anti-TNF agents in TB patients. The log-rank test indicates a significant difference between curves. preTB_antiTNF = 0: non-user with anti-TNF agents before being diagnosed with tuberculosis, preTB_antiTNF = 1: user with anti-TNF agents before being diagnosed with tuberculosis.

**Figure 2:**
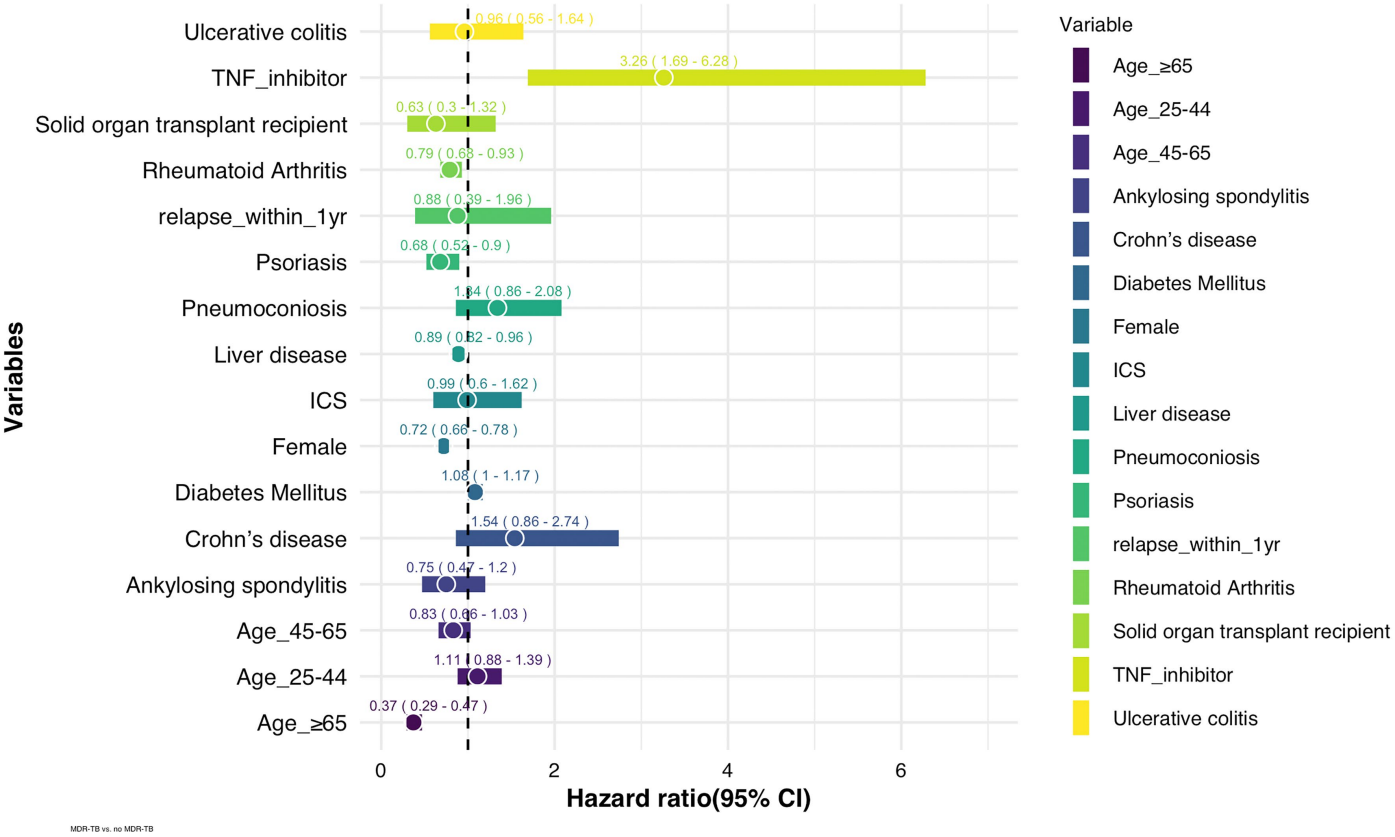
Adjusted hazard ratios of risk factors for MDR-TB in TB patients (*n* = 248,176). ICS: inhaled corticosteroid, TNF: tumor necrosis factor.

The incidence of MDR-TB was significantly higher in patients treated with anti-TNF agents than in those who were never exposed to anti-TNF agents (Figure 1). The hazard ratio (HR) of MDR-TB, adjusted for age, sex, comorbidities, and history of previous inhaled corticosteroid treatment, was 3.26 times higher in patients treated with anti-TNF agents than in those who were never exposed to anti-TNF agents (HR 3.26, 95% CI 1.69–6.28) (Figure 2). The risk for MDR-TB was also increased in males and younger patients.

Among patients who received anti-TNF agents, 43 were treated with two or more anti-TNF agents. MDR-TB was significantly more prevalent in patients who had been treated with etanercept than in those without MDR-TB (0.12% vs. 0.03%, respectively, *p* < 0.01) (Table 2). Patients with MDR-TB included a higher percentage of patients who had been treated with etanercept for rheumatoid arthritis RA than those without MDR-TB. MDR-TB was more prevalent in TB patients who received infliximab for Crohn’s disease than those without MDR-TB (0.08% vs. 0.02%, respectively, *p* < 0.01).

**Table 2:** Incidence and time to onset of muti-drug resistant pulmonary tuberculosis.

		All pulmonary TB	no MDR-TB	MDR-TB	*p*-value
Infliximab (%)		123 (0.05)	117 (0.05)	6 (0.12)	0.053
	Rheumatoid arthritis (%)	65 (0.03)	63 (0.03)	2 (0.04)	0.806
	Psoriasis (%)	21 (0.01)	20 (0.01)	1 (0.02)	0.906
	Ankylosing spondylitis (%)	29 (0.01)	28 (0.01)	1 (0.02)	1.00
	Crohn’s disease (%)	47 (0.02)	43 (0.02)	4 (0.08)	0.008
	Ulcerative colitis (%)	40 (0.02)	38 (0.02)	2 (0.04)	0.436
Interval between infliximab and TB diagnosis (days)^a^		307.0 [96.5, 576.5]	273.0 [95.0, 546.0]	1109.5 [943.5, 1264.3]	0.013
Adalimumab (%)		147 (0.06)	143 (0.06)	4 (0.08)	0.754
	Rheumatoid arthritis (%)	131 (0.05)	128 (0.05)	3 (0.06)	1
	Psoriasis (%)	22 (0.01)	21 (0.01)	0 (0.00)	0.932
	Ankylosing spondylitis (%)	61 (0.02)	59 (0.02)	2 (0.04)	0.806
	Crohn’s disease (%)	9 (0.00)	9 (0.00)	0 (0.00)	1
	Ulcerative colitis (%)	19 (0.01)	19 (0.01)	0 (0.00)	1
Interval between adalimumab and TB diagnosis (days)^a^		312.0 [167.0, 571.5]	312.0 [170.0, 576.5]	312.5 [156.8, 486.5]	0.617
Etanercept (%)		85 (0.03)	79 (0.03)	6 (0.12)	0.004
	Rheumatoid arthritis (%)	78 (0.03)	73 (0.03)	5 (0.10)	0.018
	Psoriasis (%)	16 (0.01)	16 (0.01)	0 (0.0)	1
	Ankylosing spondylitis (%)	40 (0.02)	37 (0.02)	3 (0.06)	0.057
	Crohn’s disease (%)	2 (0.00)	2 (0.00)	0 (0.00)	1
	Ulcerative colitis (%)	10 (0.00)	10 (0.00)	0 (0.00)	1
Interval between etanercept and TB diagnosis (days)^a^		812.0 [437.0, 1247.0]	812.0 [449.5, 1211.0]	898.5 [292.0, 1241.0]	0.945

MDR-TB, multidrug resistant tuberculosis.

a Data are expressed in median [interquartile range].

### Treatment outcome and adverse effects of anti-TB medication

There was no difference in the incidence of MDR-TB and no MDR-TB among patients who required retreatment 1 year after the end of treatment (MDR-TB 0.16% vs. no MDR-TB 0.19%). The MDR-TB group had significantly higher rates of hepatotoxicity (19.59% vs. 9.53%, respectively, *p* < 0.01), ototoxicity (0.26% vs. 0.02%, respectively, *p* < 0.01), optic neuropathy (2.36% vs. 1.18%, respectively, *p* < 0.01), peripheral neuropathy (5.65% vs. 4.80%, respectively, *p* < 0.01), seizures (8.79% vs. 6.79%, respectively, *p* < 0.01), and alopecia (2.64% vs. 2.04%, respectively, *p* < 0.01) after TB treatment than the non-MDR-TB group.

## Discussion

This study investigated the risk factors for the occurrence of MDR-TB in patients with pulmonary TB. As a result of multivariate analysis, younger adults and males were significantly associated with MDR-TB. While patients with Psoriasis and Rheumatoid arthritis showed low risk of MDR-TB, diabetes mellitus and solid organ transplantation showed no overt associations with MDR-TB.

We found that a prior history of treatment with anti-TNF agents was a significant risk factor for MDR-TB. Treatment with TNF inhibitors is a well-known risk factor for the reactivation or remote acquisition of TB (16).

TNF-α is known to be associated with the function of the immune system of TB patients. Some studies demonstrated that the TNF-α level in peripheral blood cells could differentiate active TB from non-TB patients and strong TNF-a responses at TB diagnosis were significantly decreased after treatment (17, 18). Interestingly, after TB treatment, the levels of TNF-α remained elevated in the sputum-positive population and in extensively drug resistant TB patients contrary to sputum- negative group and sensitive TB patients (19, 20).

TNF-α has a dual effect in TB and acts as host resistance and susceptibility factor (21, 22). Physiological levels of TNF-α have a defensive role against mycobacterial infection. TNF-α is important for phagosome activation to kill mycobacteria and granuloma formation to prevent dissemination (23). Elevated TNF-α levels in some subgroups after treatment may be indicative of the host’s immune status to control the pathogen.

Kviatcovsky et al. reported that MDR strain M inhibited TNF-α secretion influencing on neutrophil activation and ROS production, as part of evasion mechanism to persist in the host (24). Several studies found the distinct difference in immune responses and the immune cellular subpopulation between MDR TB and sensitive TB (25, 26). Drug resistant TB can occur as primary drug resistance or acquired drug resistance happened during failed treatment of drug-susceptible TB (27). In the latter cases, bacterial subpopulation known as persisters can be phenotypically tolerant to anti-TB medication. Evidence suggests drug resistant *M. TB* strains interact with phagocytes and immune cells in the lung microenvironment and modify to adapt and persist in the host (23).

Therefore, it is reasonable to assume that TNF-α inhibitors suppress cytokine expression and alter the immune microenvironment and would develop resistant TB or advanced forms of TB. Our results, in which the occurrence of MDR-TB was more frequent in the case of longer use of infliximab, support the hypothesis that the risk of developing resistant tuberculosis might increase when TNF-α is continuously suppressed.

There were many different findings from previous studies identifying risk factors for MDR-TB (4, 5, 13, 29). In the present study, the differences in MDR-TB risk between the types of anti-TNF agents were not clear, in contrast to previous studies showing that the risk for TB is higher in patients receiving anti-TNF monoclonal antibody therapy than those receiving soluble TNF receptor therapy (28, 29). Wallis et al. (30) suggested a time-varying risk for TB in patients with anti-TNF therapy with a higher early increased risk for infliximab than for etanercept. Similarly, in our study, the average duration of drug therapy before TB diagnosis was higher for etanercept than infliximab and adalimumab.

Previous studies (4, 5, 13, 31) have reported that MDR-TB risk is mainly related to previously diagnosed TB and anti-TB therapy rather than sociodemographic characteristics, such as age or sex, comorbidities such as HIV or chronic obstructive pulmonary disease (COPD), lifestyle factors such as alcohol abuse, and other TB-related characteristics, such as directly observed therapy. The results of meta-analyzes have been from pooled studies that differed in design, patient demographic characteristics, and definitions of MDR-TB, resulting in a large degree of heterogeneity (4).

Age and sex may be related to TB drug adherence. Interestingly, in our results showed that more men and more ICS users are included in non-naïve MDR-TB patients than naïve MDR -TB patients. ICS use appears to be a risk factor for MDR-TB in terms of the decreased immune response, similar to the pathogenesis of pneumonia or non-MDR-TB (4). Further research is needed to determine the correlation between ICS exposure and emergence of drug resistance in TB.

Our data showed that while rheumatoid arthritis and psoriasis were associated with lower risk for MDR-TB, the percentage of patients who had been treated with etanercept for rheumatoid arthritis RA was higher in MDR TB patients than those without MDR-TB. Due to data limitations, it was not possible to ascertain the individual’s immune status, latent TB and latent TB prophylaxis. Given the results of the United Kingdom study showing that incidence of tuberculosis varies with the severity of psoriasis, immune status including TNF-α and IL-17 -induced inflammation may affect the MDR-TB risk (32). Further research taking into account these variables may clarify the association with MDR-TB risk.

Our findings could help improve the rate of successful TB treatment and help prepare for immediate radical treatment by actively observing pulmonary TB patients with early suspicion of MDR-TB.

This study had several limitations. First, the study used the claims data from the NHI in Korea. As the administrative claims data were designed for reimbursement purposes, they do not include sensitive information for privacy reasons, such as socioeconomic status, occupation, nutritional status, or medication adherence. Second, retreatment and recurrence were excluded as index TB infections after confirmation by observation from 1–2 years to 7 years, but recurrence after infection decades ago was not confirmed due to the limitation of claims data. Third, it was also difficult to determine the number of patients with latent TB infection before using TNF-α inhibitors. In this study, the transition rate from latent TB to active infection could not be confirmed. Fourth, this claim dataset was unable to differentiate whether the development of MDR TB is because of the anti-TNF alpha agents or the immune status of the patients using anti-TNF alpha agents. Regardless of these limitations, our results could help understand anti-TNF agents’ effects as a driver of MDR-TB in patients with pulmonary TB.

## Conclusion

This study identified an increased risk for MDR-TB in patients who received TNF-α inhibitors. Therefore, successful treatment of TB requires close monitoring of patients who have received drugs that may affect TNF-related immune dysfunction.

## Data availability statement

The data analyzed in this study is subject to the following licenses/restrictions: We cannot share the administrative data of the Korean government. Requests to access these datasets should be directed to mdhong@hallym.or.kr.

## Ethics statement

The studies involving human participants were reviewed and approved by Kangwon National University Hospital Institutional Review Board (KNUH-2022–02–011). Written informed consent for participation was not required for this study in accordance with the national legislation and the institutional requirements.

## Author contributions

YH and JP contributed to the design concept, acquisition, analysis, and interpretation of data, and drafting of and revising of the manuscript critically for important intellectual content. JH and JP contributed to the design concept, interpretation of data, and revising of the manuscript critically for intellectual content. All authors contributed to the article and approved the submitted version.

## Funding

This study was supported by the National Research Foundation of Korea grant (NRF2020R1C1C1009091 and NRF2020R1A2C1011455) and the Korea Health Technology R&D Project through the Korea Health Industry Development Institute (KHIDI), funded by Korean Government (HI21C1074).

## Conflict of interest

The authors declare that the research was conducted in the absence of any commercial or financial relationships that could be construed as a potential conflict of interest.

## Publisher’s note

All claims expressed in this article are solely those of the authors and do not necessarily represent those of their affiliated organizations, or those of the publisher, the editors and the reviewers. Any product that may be evaluated in this article, or claim that may be made by its manufacturer, is not guaranteed or endorsed by the publisher.
